# The Cost-Effectiveness Analysis of Teleglaucoma Screening Device

**DOI:** 10.1371/journal.pone.0137913

**Published:** 2015-09-18

**Authors:** Sera Thomas, William Hodge, Monali Malvankar-Mehta

**Affiliations:** 1 Department of Epidemiology and Biostatistics, Schulich School of Medicine and Dentistry, Western University, London, Canada; 2 Department of Ophthalmology, Ivey Eye Institute, St. Joseph's Health Care London, London, Canada; Bascom Palmer Eye Institute, University of Miami School of Medicine;, UNITED STATES

## Abstract

Glaucoma is the leading cause of irreversible vision loss and costs the American economy $2.9 billion. Teleglaucoma remotely detects glaucoma improving access to ophthalmic care in rural areas. It helps manage glaucoma more efficiently to preserve vision and reduce healthcare costs. A cost-effectiveness analysis was conducted using healthcare provider or third-party payer perspective within rural Canada. The study population were patients at-risk of glaucoma which includes those with diabetes and/or hypertension, family history of glaucoma, adults older than 50 years, and concurrent ocular conditions in rural Alberta. Markov modelling was used to model glaucoma health states. Effectiveness was measured in Quality-Adjusted Life Years (QALYs) and costs were used in Canadian dollars. Using TreeAge Pro 2009, incremental cost-effectiveness ratios (ICER) were developed in dollars per QALYs. Deterministic and probabilistic sensitivity analyses were performed to assess the factors affecting cost-effectiveness. Teleglaucoma had a 20% increase in ophthalmologist-referral rate; it reduced patient travel times by 61 hours and physician wait times by 30% in comparison to in-person examination (standard of care). Teleglaucoma costs $872 per patient screened which was 80% less than in-person examination. Teleglaucoma had a greater incremental effectiveness providing an additional 0.12 QALY per patient examination. It was more sensitive (86.5%) and less specific (78.6%) than in-person examination. Teleglaucoma was more cost-effective than in-person examination with an ICER of-$27,460/QALY. This indicated that teleglaucoma will save $27, 460 for each additional QALY gained. Long term benefits showed teleglaucoma prevents 24% cases of glaucoma blindness after 30 years. Teleglaucoma demonstrated improved health outcomes, as well as, cost benefits. It increases access to ophthalmic care and improves healthcare service efficiency, specifically in rural areas. Teleglaucoma is more cost-effective than current in-person examination and can improve the quality of life in glaucoma patients.

## Introduction

The burden of vision loss on the Canadian economy is $15.8 billion per year in which 55% is allocated to direct health care costs [1]. Sixty-five per cent of adults with partial or full vision loss are unemployed, which translates to $4.06 CAN billion annually of lost earnings [1]. In the United States, vision loss costs over $35 billion for direct costs and loss of productivity [2]. Glaucoma is the major eye disease leading to irreversible vision loss. The economic burden of glaucoma alone on the American economy is $2.9 billion [2].

Glaucoma is an age-related disease affecting the elderly at higher rates. An estimated 3% of the global population over 40 years of age currently has glaucoma, the majority of whom are undiagnosed [3]. The global prevalence of glaucoma is increasing and with the growing elderly population and current management, it is expected that 79.6 million people will have glaucoma by 2020 [3].

Glaucoma tends to be detected at later stages of the disease when glaucoma has advanced into vision impairment. Glaucoma is characterized by distinctive peripheral visual field loss. Patients have “tunnel vision” but may have perfect central vision. As a result, patients may not notice visual field loss until advance stages of disease. Detection of glaucoma at earlier stages is important for treatment and to prevent the progression of disease [4].

Teleglaucoma is the application of electronic technologies to ophthalmic instruments to identify glaucoma cases and those at-risk of glaucoma [5]. Teleglaucoma remotely detects glaucoma via electronic transmission of high-resolution stereoscopic fundus photographs. It involves one or more graders who conduct the ophthalmic tests, read the results, and send the reports to the ophthalmologist [6]. Teleglaucoma is hypothesized as a more efficient way of managing glaucoma in rural areas, such as Alberta. The University of Alberta provides remote and in-house teleglaucoma services for rural Alberta residents where patients undergo a standardized interview to collect medical history information [6].

Currently this technology is validated for use in diabetic retinopathy, but recent research has assessed its performance for glaucoma [7]. Several studies have reviewed the effectiveness of teleglaucoma: Li et al. found moderate agreement between digital optic nerve assessments and slide films [8]. Another study reported good correlation between cup-to-disc ratios from teleglaucoma and ophthalmoscopy [9]. It was reported by Tuulonen et al that patients were satisfied with teleglaucoma service as it successfully reduced patient costs by 92%, saved patient time by 92%, and there was a 97% reduction in patient travel [10]. Teleglaucoma also reduces the patient load in ophthalmic clinics; a study by Verma et al found that the majority of teleglaucoma patients did not require in-person consultation and could be managed with teleglaucoma [11]. A recent study by Thomas et al synthesized the effectiveness of teleglaucoma and found teleglaucoma was effective at screening negative cases [7]. The technology gave poor quality images in only 10.4% of images [7]. It improved access to ophthalmologist and had a referral rate of 12.5% to the ophthalmologist [7].

With implementation of any new technology and service comes an additional cost. Thomas et al reported that teleglaucoma had a mean cost per patient screened of $922.77 (US) and a mean cost per detected case of $1098.67 (US) [7]. However, there are no economic evaluations in literature which examine the cost-effectiveness of the use of telemedicine for glaucoma. Thus, the purpose of this cost-effectiveness analysis (CEA) was to examine the costs and benefits of teleglaucoma and to determine the cost-effectiveness of teleglaucoma as a screening device for glaucoma in comparison to the standard of care, which is in-person examination. This CEA took a third-party payer and Ministry of Health perspective. The targeted population was people living in rural Alberta who are at-risk of glaucoma. The long term benefits of teleglaucoma including prevention of vision loss from glaucoma was also assessed.

## Methods

### Study Design

A cost-effectiveness analysis was conducted using healthcare provider perspectives within rural Alberta, Canada. Statistics Canada defines rural populations as areas with persons living outside centres with a population of 10,000 or fewer and outside areas with fewer than 400 persons per square kilometre [12]. Other than certain parts of Edmonton and Calgary, the majority of communities in Alberta are rural areas. It has been documented that 95% of Alberta is rural area [13].The study population are patients at-risk of glaucoma which includes those with diabetes and/or hypertension, family history of glaucoma, older adults, and concurrent ocular conditions in rural Alberta. Targeting at-risk populations has been suggested as a more efficient method of detecting glaucoma [14]. Teleglaucoma screening in the model was applied to a population aged 50 years and older at a frequency of one screening per year. The model assumed teleglaucoma has the capacity for 300 people per year [10]. The time horizon was 30 years as glaucoma is a chronic, life-long condition.

### Markov Model

Markov modelling was used to model glaucoma health states (mild, moderate, severe, and blind). The model assumed that patients who were screened correctly as glaucoma positive with either teleglaucoma or in-person examination received standard of care treatment. Different pathways of treatment were not included in the model. Using TreeAge Pro 2009, incremental cost-effectiveness ratios (ICER) were developed in dollars per Quality Adjusted Life Years (QALYs). Effectiveness was measured in QALYs and costs were used in Canadian dollars. The cycle for the Markov model represented one year and the ICERs following 30 cycles were established. Additional model parameters are listed in S1 File.

### Health States

There are four health states associated with glaucoma: mild, moderate, severe, and end-stage glaucoma which is blindness. Mild glaucoma is characterized by abnormalities of the optic nerve without any visual field abnormalities. Moderate glaucoma is characterized by damage to the optic nerve and some peripheral vision loss. Severe glaucoma is the advanced stage of glaucoma characterized by severe optic nerve damage and advanced peripheral vision loss. Blindness is characterized by a visual acuity of 20/200 or worst [15]. Blindness in this study refers only to blindness due to glaucoma.

Glaucoma is a chronic condition with progressive ocular damage and vision loss. Patients will progress from one stage to the next and with successful treatments the patient will remain in the current health state. There is no cure for glaucoma and thus patients cannot transition to healthier states. Once a patient is blind, the patient will remain blind.

### Utility Values

Utility values were used to measure the quality of life associated with each health state. Utility values are an economic measure that quantifies quality of life from 1 meaning perfect health to 0 meaning poor health or death. Utility values were converted into QALYs as the standard unit for cost-effectiveness analyses are $/QALY. Cost-effectiveness analysis use $/QALY because it a universal unit which allows comparison of ophthalmic and non-ophthalmic interventions and their cost-effectiveness. QALY incorporates both quantitative and qualitative and it adjusts life expectancy based on the quality of life. It applies weights on different health states. For example, the utility value associated with being blind is 0.5 which means one year living in blind state is equivalent to half a year living in perfect health (utility value = 1.0). In accordance, the QALYs associated with 1 year living in blind state is 0.5 QALYs.

### Costs

There are three main components of teleglaucoma that each are associated with costs: human resources, information technology, and diagnostic equipment (Table 1) [7]. The synthesis of teleglaucoma costs derived by Thomas et al and the Ministry of Health Medical Procedures List were used as costing data sources [7], [16]. Costs reported in the literature in euros or other currencies had to be converted to one standard currency to allow for consistency when totalling costs. Costs which were reported in Euros were converted to 2014 Canadian dollars and adjusted for inflation at 2.05% [7].

**Table 1:** Standardized Teleglaucoma Equipment.

Human Resources	Information Technology	Screening Equipment	Examinations
Graders	Videoconferencing equipment,	Retinal camera, Tonometer	Medical & family history
Physicians/ ophthalmologists	Secure Diagnostic Imaging (SDI) system,	Devices to measure central corneal thickness	Visual acuity
Glaucoma Specialists	Computer systems and software	Frequency Doubling Technology (FDT) or Humphrey Visual Field test	Pupil equal and reactive to light (PERL) or relative afferent pupillary defect (RAPD)
Ophthalmic technicians		Optical Coherence Tomography	CCT
		Slit lamp, Gonioscope	OCT
		Retinal camera	Slit lamp
			Gonioscopy
			Visual field
			Ancillary tests
			Fundus photographs
			IOP

Citation: Thomas S-M, Jeyaraman MM, Hodge WG, Hutnik C, Costella J, Malvankar-Mehta MS. (2014) The Effectiveness of Teleglaucoma versus In-person Examination for Glaucoma Screening: A Systematic Review and Meta-Analysis. PLoS ONE 9(12): e113779. doi: 10.1371/journal.pone.0113779. pmid:25479593

Costs were divided by the number of patients serviced to determine the costs per patient and also to account for the differences in coverage between in-person care and teleglaucoma. Teleglaucoma was reported to service 300 people per year, while in-person care was reported to have 1379 glaucoma visits per year in rural Alberta [8],[17]. Teleglaucoma requires training of graders on how to use the technology. The costs for training includes labour costs for two (full-time equivalents) trainers at the average Alberta salary ($50,000) and training resources [18]. The direct costs of teleglaucoma included the costs of equipment, set-up, overhead, utilities, and labour. The costs of equipment was sourced from vendor quotes reported by Thomas et al [7]. The costs of labour for in-person examination was sourced from the Alberta Provincial Medical Procedures List [16]. Sensitivity analysis was applied to accommodate the effect of the above costing assumptions and its effects on the results.

Each health state requires different levels of medical treatments and drug therapies. In addition, each is associated with indirect costs such as health system costs, loss of productivity, additional vision aids, and modifications to home or work to compensate for vision loss. The costs associated with each health state was given by Lee et al. study on resource consumption at different levels of severity of glaucoma [19].

The stage at-risk was assumed to be equal to “Stage 0” of Lee’s criteria which constitutes glaucoma suspect patient who is at-risk of glaucoma but does not meet criteria for clinical diagnosis [19]. The costs associated with at-risk includes routine optometrists and/or ophthalmologist visits. The costs of blindness were reported by the Canadian National Institute for the Blind (CNIB) [1], [20]. The costs of blindness includes direct costs (vision aids and treatments) as well as indirect costs such as loss of productivity, caregiving assistance, etc.

All costs were summed into initial and incremental costs and cost per patient screened was determined. The initial costs were the fixed costs such as the initial set up fees. The incremental costs included the patient costs, service costs, labour costs and costs associated with each health state. Costs were converted to present value Canadian dollars and future costs were discounted at a 3% rate. The willingness to pay applied was $40,000/QALY as reported by literature for ophthalmic interventions [15]. Uncertainties in estimated costs were addressed using probabilistic sensitivity analysis and applying gamma distributions.

### Analysis

This study analyzed the incremental costs, the incremental effect, and the ICER for teleglaucoma versus in-person examination. Deterministic and probabilistic sensitivity analyses were performed to assess the factors affecting cost-effectiveness. Markov Cohort Analysis by 30 stages was conducted to demonstrate the accumulated rewards, costs, and probabilities after 30 years. The default for Markov Cohort Analysis in TreeAge Pro software is 30 stages. Monte Carlo Simulations with the application of second-order uncertainties with gamma and beta distributions was performed with 1000 samples. In addition, the analysis generated the distribution of the ICERs by probability, the cost-effectiveness scatterplots, and the impact of willingness-to-pay on the probability of ICERs within an acceptability curve.

## Results

The model parameters for the effectiveness measures and utility measures were as follows:

### Effectiveness

Cited from ophthalmic research literature, the specificity and sensitivity of teleglaucoma were 86.5% and 78.6% respectively [7]. The effectiveness of teleglaucoma was also measured in its reduction of travel time and improved access to care for people living in rural Alberta and other remote, underserviced areas. Specifically, teleglaucoma has been associated with savings of 4906km in travel distance and 61.23 hours of travelling time [21]. The length of time spent at the doctor visit (includes wait time and assessment time) with teleglaucoma was 78 minutes (~1.3 hours) whereas with in-person care it took 115 minutes (~1.91hours) [22].

### Utilities

The utility value for each health state was 0.87, 0.79, 0.64, and 0.5, for mild, moderate, severe, and blindness, respectively [23], [24]. These values were converted to QALYs, as the ultimate unit of effectiveness for the cost-effectiveness analysis. The QALYs associated with the following glaucoma health states: mild, moderate, and severe glaucoma are 0.87 QALYs, 0.79 QALYs, and 0.64 QALYs, respectfully.

### Incremental Cost-Effectiveness Ratio

The ICER for teleglaucoma screening versus in-person screening (no-screening) was established in *TreeAge 2009* displaying the ratio of incremental costs (Canadian dollars) and incremental effectiveness (QALYs) at a discounted rate of 3% (Table 2).

**Table 2:** Summary of ICER Data.

Strategy	Cost	Incremental Cost	Effect	Incremental Effect	Cost/Effect	ICER
Teleglaucoma Screening	871.54		18.32		47.57	
Inpatient Screening	4441.42	3569.88	18.19	-0.12	244.05	(Dominated)

**Fig 1:**
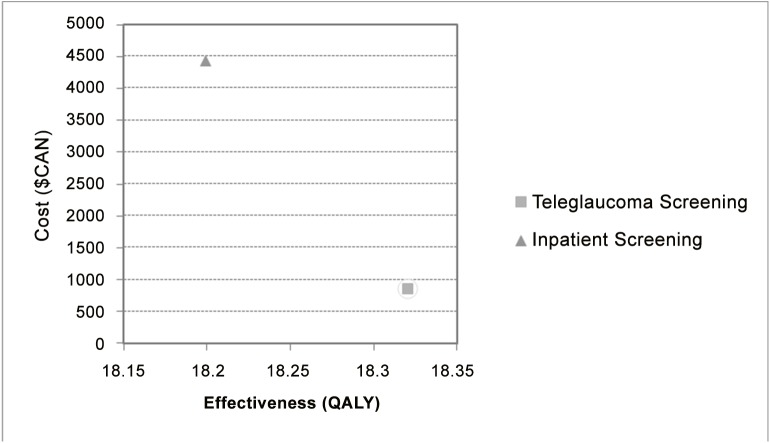
Cost-Effectiveness Analysis.

Teleglaucoma demonstrated to be more cost-effective than in-person care for detecting glaucoma; the ICER was $47.60/QALY. This means that spending an additional $47.60 for each patient screened with teleglaucoma will give an additional QALY in comparison to in-person screening. The results also indicated that teleglaucoma costs less than in-person screening when adjusted for per patient costs and also was more effective. Thus, the no screening option (in-person examination) is dominated by teleglaucoma screening (Fig 1). In most cases, cost-effectiveness analysis are not performed under these conditions (more effective, less costly). However, this study included long-term effectiveness, which was not investigated previously in literature and thus this analysis has established new information.

### Markov Cohort Analysis

Based on markov model principles, transitional probabilities are independent of previous health states and they determine the proportion of individuals who transition to other heath states per cycle [25]. Markov Cohort Analysis was conducted with 30 cycles representing 30 years. The default for Markov Cohort Analysis is 30 cycles.

After 30 years, teleglaucoma showed rewards for people with glaucoma who were initially screened positive. The total reward for teleglaucoma was 15.7 QALYs which was 1.1 less than rewards from in-person care (Table 3). However, the cumulative costs per patient for in-person care was almost 3.5 times that of teleglaucoma after 30 years, which indicated the cost-saving associated with teleglaucoma screening. For both interventions, after 30 years the majority of patients were blind, however it was 24% less in teleglaucoma screening. Teleglaucoma also had a greater probability of preventing glaucoma patients from progressing as 15% were in mild state compared to 2% with no screening.

**Table 3:** Accumulate Rewards, Costs, and Probabilities after 30 years.

					Probability at each health state		
	Cumulative Costs ($)	Cumulative Rewards ($)	At-Risk	Mild	Moderate	Severe	Blind
Teleglaucoma	1155.45	15.7	3.71E-05	0.15	0.1	0.09	0.65
In-person/ no screening	4035.19	16.8	0				

**Fig 2:**
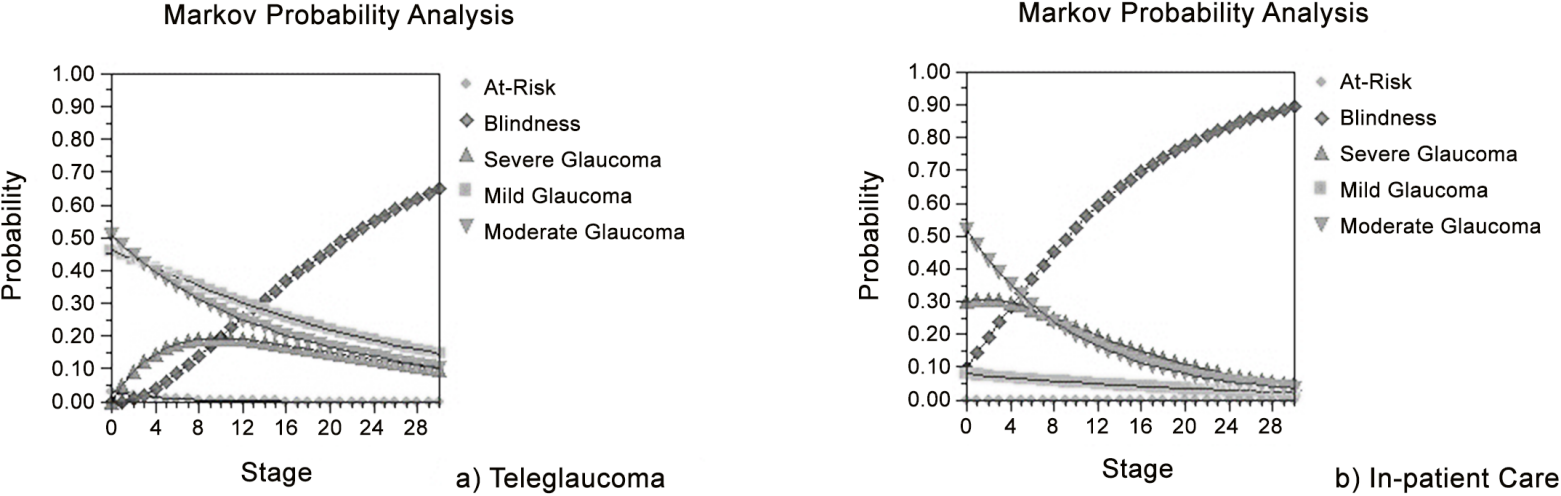
Markov Probability Analysis of Health States.

The Markov Probability Analysis displayed how the probability of each health state changes over the study time horizon in patients who were detected positive for glaucoma with either intervention (Fig 2). The results demonstrated that the probability of being at-risk for glaucoma and moderate glaucoma over 30 years (30 stages) remains relatively the same in teleglaucoma versus in-person care. The probability of being in mild glaucoma is higher with teleglaucoma screening but in both interventions this probability declines with time. The probability of being blind was greater with in-person care than with teleglaucoma (the concave down increasing trend of the blind state curve in Fig 2B displays a closely exponential trend). This indicated that teleglaucoma is more effective at preventing the probability of blindness in glaucoma patients.

### Deterministic Sensitivity Analysis

**Fig 3:**
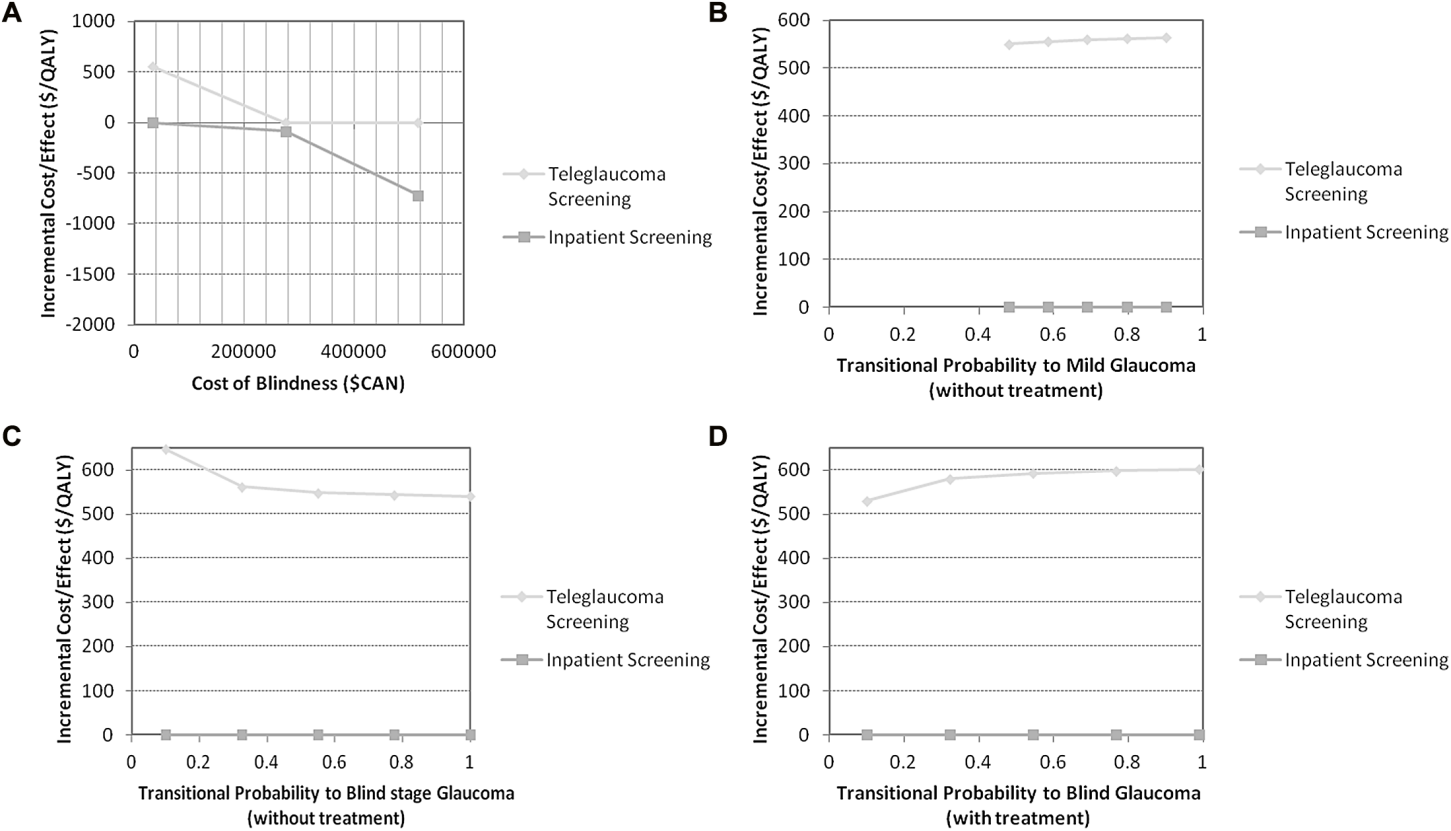
DSA One-Way Sensitivity Analysis.

Deterministic sensitivity analysis was used to determine the effects of uncertainty on the ICER results. One-way sensitivity analyses were performed on the following variables: the costs of blindness, the transitional probabilities for at-risk to mild glaucoma and severe glaucoma to blind states (with and without treatment). The results demonstrated that changing (+/- 20%) the costs of blindness caused changes in the ICERs for both strategies. Teleglaucoma had higher ICERs than inpatient screening (Fig 3). The cost-effectiveness of teleglaucoma is affected by the costs of blindness: as costs of blindness increases the ICER for teleglaucoma becomes smaller.

As shown in Fig 3D, the ICERs of inpatient screening remained unchanged while the ICER of teleglaucoma increase very slightly as the transitional probability of blindness increased. With better treatment of glaucoma which prevents patients from becoming blind, teleglaucoma becomes more cost-effective (Fig 3D).

**Fig 4:**
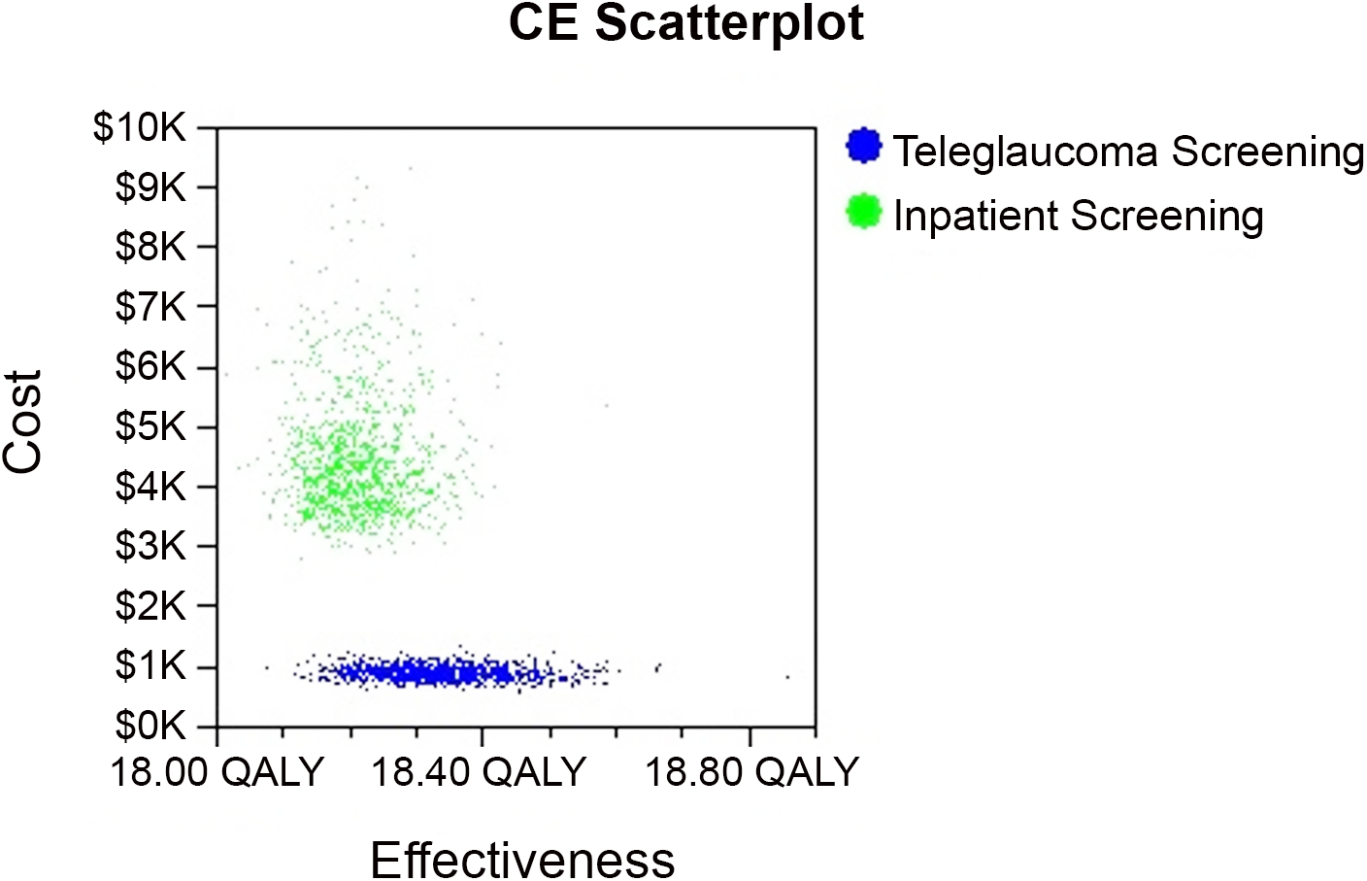
Cost-Effectiveness Scatterplot.

The tornado diagram gives the parameters with the most effect on cost-effectiveness at a willingness to pay of $40,000/QALY (Fig 4). It displays that the uncertainty within the prevalence of glaucoma has the most effect on the ICER and it has the largest range of net monetary benefits. The results suggest the transitional probabilities for at-risk to mild and severe to blind have more of an effect on the cost-effectiveness of teleglaucoma as well as the cost of blindness. Whereas, the transitional probability for severe to blind without treatment and at-risk to mild with treatment had less effect on the cost-effectiveness of teleglaucoma.

### Probabilistic Sensitivity Analysis

Gamma and beta distributions were applied to the Markov model. Monte Carlo Simulation second order was conducted and the statistics report gave a mean cost of teleglaucoma as $866.90 ± 113.10 per patient screened compared to in-person screening which has a mean of $4419.8 ± 1044.70. The results showed teleglaucoma costs less per patient than in-person screening.

**Fig 5:**
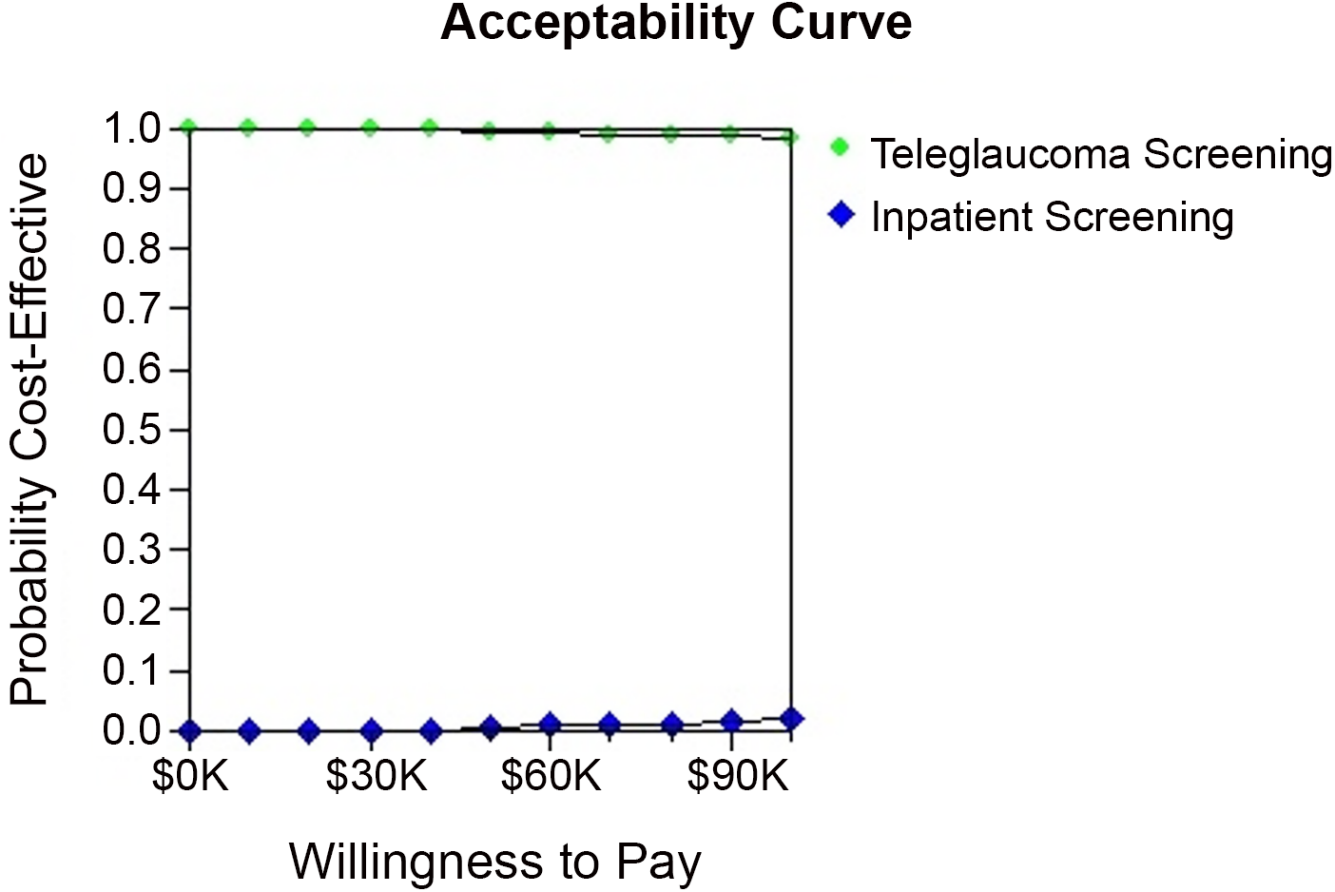
Acceptability Curve.

The results of the Cost-effectiveness scatterplot demonstrate that there is a greater uncertainty with the costs and effectiveness of “in-person screening” (in-person care) as the dots of the graph are widely spread apart giving costs from approximately $3K-8K (Fig 4). However, there is less uncertainty with the costs of teleglaucoma the dots are tightly plotted around $1K (Fig 4). This means that the ICER of in-person care is more sensitive to the costs than the ICER of teleglaucoma whereas teleglaucoma ICER is more sensitive to the effectiveness in comparison to in-person care. The results of the sensitivity analysis on willingness to pay (WTP) demonstrate that neither teleglaucoma nor in-person care is sensitive to changes in WTP as the line remains relatively constant as WTP changes (Fig 5). Only after WTP increases above $60,000, the probability of cost-effectiveness for teleglaucoma becomes slightly less cost-effective versus in-person screening which becomes slightly more cost-effective. However, in comparison to in-person screening teleglaucoma is 100% more cost-effective.

## Discussion

Teleglaucoma is beneficial to remote areas as the physician is not required to see patients in person which reduces wait times and shortens the length of ophthalmic consultations. Teleglaucoma avoids long distance travel and time wasted on commute. Our results demonstrated the direct benefits to patients was a cost savings of ~$2474.60 with teleglaucoma. The early detection approach of teleglaucoma successfully reduced the probability of patients in blind state by 24% and maintained 13% more patients at mild stage glaucoma in comparison to in-person care. The long-term benefits of early detection was confirmed by this CEA with greater cumulative rewards and cost savings 30 years post-detection. When assessed on its own, teleglaucoma was more cost-effective than in-person care with an ICER of-$27,460 per QALY (cost per patient serviced) meaning teleglaucoma saved $27,460 per QALY gained relative to in-person examination. The large direct patient savings and reduced costs of blindness due to preservation of vision, mainly accounted for its effectiveness. The ICER of teleglaucoma was only sensitive to the probability of glaucoma. This is logical since positive predictive values of screening tools fluctuate with changing prevalence rates and changing prevalence rates will alter the probability of glaucoma. As the probability of having glaucoma increases, teleglaucoma had greater cost-effectiveness.

At a willingness to pay of $40,000/QALY, teleglaucoma is cost-effective when compared with in-person care [20]. In addition, the World Health Organization provides the threshold for cost-effective interventions: an intervention is considered cost-effective if the ICER associated with implementation of the intervention is less than the country’s GDP [26]. Teleglaucoma has an ICER below Alberta’s GDP and thus, teleglaucoma is cost-effective for Alberta’s population.

Several studies have analyzed the effectiveness of teleglaucoma in terms of its ability to detect glaucoma and proposed reduction in direct patient costs, however, none have produced a complete cost-effectiveness analysis [5–9]. Analysis of teleophthalmology for other ocular conditions such as diabetic retinopathy, have also shown to be cost-effective with ICERs of $1320/QALY in a similar rural setting based on the data of 326 patients from rural India [27].

Teleglaucoma also provides opportunities for collaborative care to recognize the multi-faceted nature of glaucoma management and to optimize patient health outcomes overall. Kassam et al proposed a “shared care pathway” for teleglaucoma which emphasized patient-centred glaucoma management [28]. It involved a collaborative care model between the primary-care provider, the optometrist, and the ophthalmologist who share health information amongst each other to better manage the patient [28]. This model has the potential improve healthcare efficiency and to reduce costs. With additional healthcare providers involved, there would be increased salary costs associated with collaborative care. However, there is potential for greater patient health benefits than standard teleglaucoma service. Thus, the benefits will outweigh the costs, and potentially teleglaucoma under a collaborative care model would be a cost-effective healthcare approach.

The strength of this study is it indicated that although the base costs of teleglaucoma are large, the variable costs are lower per year, in that the benefits outweigh costs over time. In addition, this study includes indirect costs such as loss of productivity and opportunity costs of time. By including a patient, healthcare provider, as well as the Ministry of Alberta perspective, a societal perspective is developed providing a broad scope on the cost-effectiveness of teleglaucoma. This CEA is focused on screening for a targeted population who is above age 50 years and at-risk of glaucoma in rural Alberta which is a strength. Mass screening of total populations are not cost-effective as it wastes resources with small benefits. In addition, this CEA applied Markov Modelling to illustrate the progression of glaucoma through transitional health states over time. This is beneficial to predict the long-term benefits of teleglaucoma. Costs were also discounted at a 3.0% rate to account for future value. Most studies have reported only the patient’s present benefits at time of the teleglaucoma screening, but have not analyzed the aftermath. Thus, with a time horizon of 30 years this CEA contributes to literature by illustrating early detection with teleglaucoma delays the progression of glaucoma and preserves vision.

One of the limitations within the CEA is that because no studies have analyzed the long-term benefits of teleglaucoma, estimates of transitional probabilities were derived from non-teleglaucoma studies. The Monte Carlo simulation applied several assumptions which should be taken into consideration when interpreting the results. The model assumed all screen positive cases received treatment with the standard of care. Due to uncertainties in treatment pathways, the variation in treatment paradigms, the effectiveness of treatments, and the heterogeneity of patient risk factors and outcomes, it is challenging to generate an accurate model of treatment pathways, thus teleglaucoma was accessed for screening purposes only. It is important to note that the Monte Carlo is a simulation model made to simulate reality as close as possible, but based on the inputs from literature. Thus, the long term benefits of teleglaucoma reported through modelling, like any other modelling, should be interpreted with caution.

In addition, there is a lack of RCT data on teleglaucoma as most studies are observational. Of the observational studies that did look at effectiveness of teleglaucoma, most focused on diagnostic accuracy, patient satisfaction, and reduced patient costs, but did not examine clinically relevant outcomes such as reduction in patients with vision loss.

In conclusion, a cost-effectiveness analysis of teleglaucoma was successfully performed to demonstrate that implementing teleglaucoma in rural Alberta and targeting at-risk population is cost-effective in comparison to no screening. Early detection of glaucoma allows necessary medical care to prevent progression of disease. Glaucoma is a chronic progressive disease with no cure and thus this CEA provides valuable prognosis information. Teleglaucoma can have long-term benefits on preservation of vision in those with glaucoma.

## Supporting Information

S1 File Model Parameters. (DOCX)
