# Supplementary material for: The Cost-Effectiveness Analysis of Teleglaucoma Screening Device
Source: PLoS One. 2015 Sep 18;10(9):e0137913. doi: 10.1371/journal.pone.0137913 (PMC4575061; doi:10.1371/journal.pone.0137913)
Supplement: S1 File — (DOCX) [file pone.0137913.s001.docx]

S1 File: Model Parameters

Transitional Probabilities for Glaucoma Health States

|  | Health States | Transitional Probability | Sensitivity Analysis Range |
| --- | --- | --- | --- |
| Treated | At-Risk to Mild Glaucoma | 0.20 | 0.16-0.24 |
|  | Mild to Moderate Glaucoma | 0.04 |  |
|  | Moderate to Severe Glaucoma | 0.10 |  |
|  | Severe Glaucoma to Blind | 0.15 | 0.12-0.18 |
| Untreated | At-Risk to Mild Glaucoma | 0.48 | 0.38-0.58 |
|  | Mild to Moderate Glaucoma | 0.26 |  |
|  | Moderate to Severe Glaucoma | 0.5 |  |
|  | Severe Glaucoma to Blind | 0.5 | 0.4-0.6 |

Direct Costs of Teleglaucoma and in-person Care

|  | **Teleglaucoma** | **in-person** **Care** |
| --- | --- | --- |
|  | **Total Fixed Costs ($)** | |
| Set-up (Service and Training) | 416,600 | 243,146 |
| Technology Equipment | 1,256,142 | 329,833 |
|  | **Variable Costs ( $ costs per patient screened)** | |
| Labour | 348 | 248.98 |
| Service | 370.89 | 309.90 |

Costs associated with each health state

| Health State | Costs ($) | Sensitivity Analysis |
| --- | --- | --- |
| At-Risk | 623 |  |
| Mild Glaucoma | 1480 |  |
| Moderate Glaucoma | 3682 |  |
| Severe Glaucoma | 4975 |  |
| Blindness | 33666 | 26,932.80 - 40399.20 |

Probabilities of glaucoma detection and associated health states

|  | **Probability of Being Detection** | | **Probability of each health state detected** | | | | |
| --- | --- | --- | --- | --- | --- | --- | --- |
|  | Sensitivity | Specificity | At-risk | Mild | Moderate | Severe | Blind |
| Teleglaucoma | 0.59 | 0.96 | 0.03 | 0.46 | 0.5099 | 0.0001 | 0 |
| in-person care | 0.50 | 0.50 | 0 | 0.08 | 0.52 | 0.30 | 0.1 |

Citation: Thomas, Sera, "The Effectiveness of Teleglaucoma versus In-patient Examination. Assessment: Systematic Review, Meta-Analysis, and Cost-Effectiveness Analysis" (2015). Electronic Thesis and Dissertation Repository. Paper 2934.
http://ir.lib.uwo.ca/etd/2934
